# Supplementary material for: Evidence supported by Mendelian randomization: impact on inflammatory factors in knee osteoarthritis
Source: Front Med (Lausanne). 2024 May 28;11:1382836. doi: 10.3389/fmed.2024.1382836 (PMC11165061; doi:10.3389/fmed.2024.1382836)
Supplement: Supplementary file 1 [file Data_Sheet_1.ZIP › data/GCST90274795_5e6/795.forest.pdf]

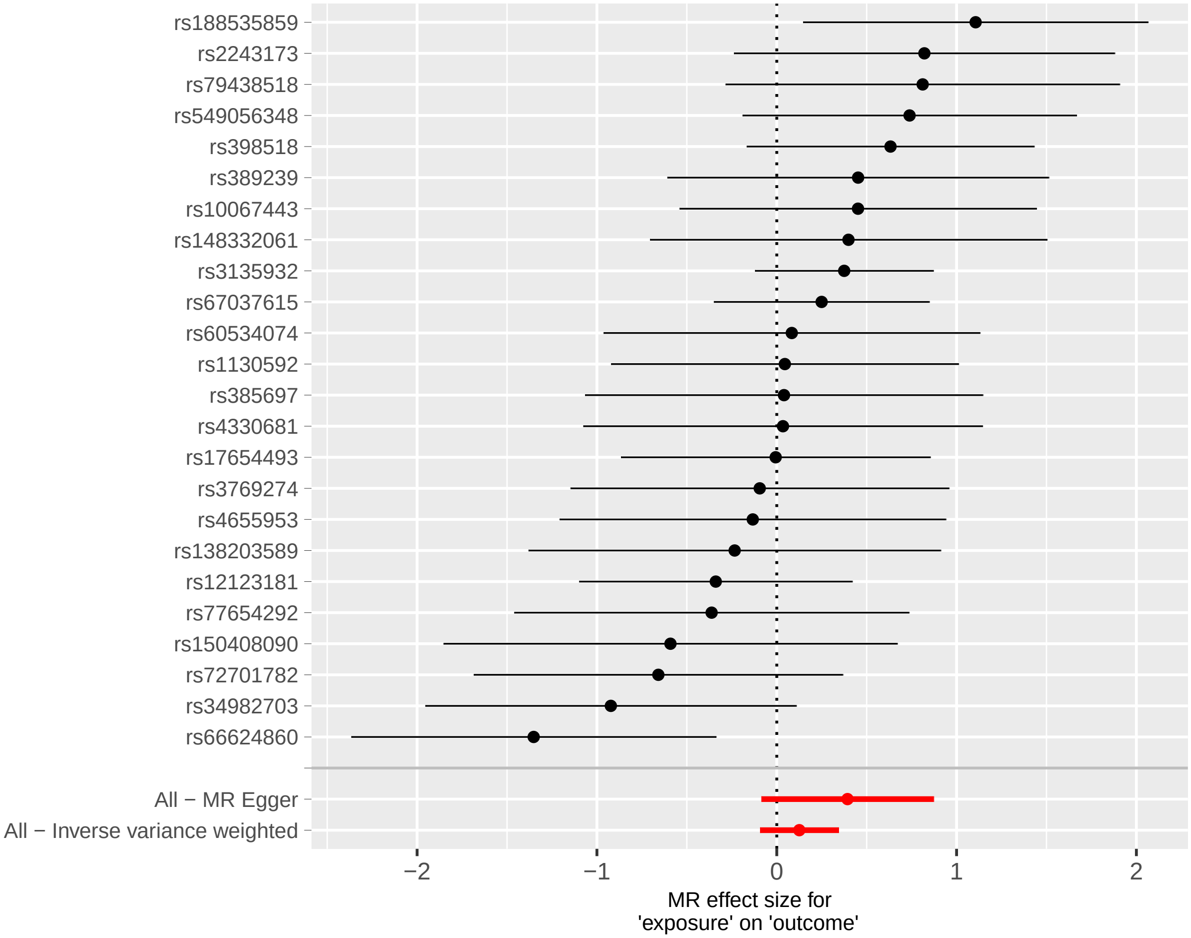

rs188535859
rs2243173
rs79438518
rs549056348
rs398518
rs389239
rs10067443
rs148332061
rs3135932
rs67037615
rs60534074
rs1130592
rs385697
rs4330681
rs17654493
rs3769274
rs4655953
rs138203589
rs12123181
rs77654292
rs150408090
rs72701782
rs34982703
rs66624860
All − MR Egger
All − Inverse variance weighted
−2
−1
0
1
2
MR effect size for
'exposure' on 'outcome'
